# Reaching Perinatal Women Online: The *Healthy You, Healthy Baby* Website and App

**DOI:** 10.1155/2014/573928

**Published:** 2014-04-28

**Authors:** Lydia Hearn, Margaret Miller, Leanne Lester

**Affiliations:** ^1^School of Dentistry, University of Western Australia, Perth, WA 6009, Australia; ^2^School of Exercise and Health Science, Edith Cowan University, Joondalup, WA 6027, Australia; ^3^Child Health Promotion Research Centre, Edith Cowan University, Joondalup, WA 6027, Australia; ^4^Health Promotion Evaluation Unit, University of Western Australia, Perth, WA 6009, Australia

## Abstract

Overwhelming evidence reveals the close link between unwarranted weight gain among childbearing women and childhood adiposity. Yet current barriers limit the capacity of perinatal health care providers (PHCPs) to offer healthy lifestyle counselling. In response, today's Internet savvy women are turning to online resources to access health information, with the potential of revolutionising health services by enabling PHCPs to guide women to appropriate online resources. This paper presents the findings of a project designed to develop an online resource to promote healthy lifestyles during the perinatal period. The methodology involved focus groups and interviews with perinatal women and PHCPs to determine what online information was needed, in what form, and how best it should be presented. The outcome was the development of the Healthy You, Healthy Baby website and smartphone app. This clinically-endorsed, interactive online resource provides perinatal women with a personalised tool to track their weight, diet, physical activity, emotional wellbeing, and sleep patterns based on the developmental stage of their child with links to quality-assured information. One year since the launch of the online resource, data indicates it provides a low-cost intervention delivered across most geographic and socioeconomic strata without additional demands on health service staff.

## 1. Introduction

Overwhelming evidence reveals close links between maternal obesity, excess gestational weight gain, unhealthy maternal lifestyles, and childhood adiposity [1]. High maternal BMI and excess weight gain are independently predictive of high birth weight and increased child and adolescent overweight and adiposity [2]. Dual mechanisms both of metabolic imprinting by maternal diet and obesity during pregnancy [3] and family lifestyle influences in early childhood have been proposed [2]. Educating and supporting mothers to achieve healthy lifestyles and weight in the perinatal period is therefore critical to preventing child obesity [3].

In Australia, over 46% of women are either overweight or obese at the start of pregnancy, and one-third gain excessive weight during pregnancy [4, 5]. Retention of extra weight postpartum is also common [6]. By the age of three years, 20% of Australian children are overweight or obese [7], with their weight closely linked to that of their mothers [8]. Not only has the prevalence of overweight and obesity increased in Australia, but the distribution of BMI has also shifted towards the upper end for parents and their children [9].

Recent meta-analytical studies indicate the positive outcomes of interventions to prevent overweight and obesity during pregnancy [10, 11]. Perinatal health care providers (PHCPs) increasingly acknowledge the importance of promoting healthy weight during childbearing years, yet, in practice, time, remuneration, knowledge, skills, and capacity hamper their ability to provide mothers with counselling and advice [12, 13]. Furthermore, maintaining healthy lifestyle behaviours is challenging for pregnant women and new mothers today as they confront competing family and work demands, fatigue, time pressure, lack of motivation, and reduced support from family and others due to dispersed living arrangements [14].

In response, mothers increasingly rely on online resources to access health related information 24 hours a day, with instant answers to their questions and concerns, and diverse advice from which mothers can choose what suits them [14–22]. In the past, PHCPs were a primary source of health information for new mothers, providing consultation, educational pamphlets, and parent classes [23]. Today PHCPs need to be ready to support pregnant women with interpretation and application of information retrieved online [10, 17, 24].

Australia has the fifth highest internet use per person in the world, with over 92% of Australians having home internet connection [25] and women of childbearing age amongst the group with highest access to household broadband [26]. Mobile wireless internet connection is the fastest growing online resource, particularly among this younger generation [25], with a 32% increase in the volume of data downloaded via mobile handsets in the past year [26]. Key benefits to PHCPs of online health information for new mothers include the ability to reach a wider audience without increase in service costs [27–29], as well as the opportunity for health promotion initiatives to engage with individuals wherever and whenever needed.

In addition to static website information, mothers are using interactive online environments such as Facebook, blogs, and smartphone applications (apps), which provide important social support especially when women are isolated, time poor, or needing reassurance [21]. To this end, young women seek participation in electronic support groups for information and advice to navigate and deal with challenges during and after pregnancy [20, 21, 30]. Research indicates that approaches using personalised online information with tailored messages [29, 31] are more effective in achieving longer-lasting health behaviour change than static information [32–34], yet little research has been conducted to better understand what online information mothers need during the perinatal period and how to make it useful and appealing [35].

If PHCPs are to benefit from the opportunities provided by the “virtual” environment as positive means of addressing the obesity epidemic among perinatal mothers and their offspring, then research is required to identify what online information young women feel is most needed, in what format, and how best it should be presented [22]. Although numerous websites and apps are emerging to reach perinatal women [21, 36] rarely has research to inform the design and planning of these online resources been reported.

This paper summarises the findings of a study to determine online information needs of perinatal women regarding healthy eating, physical activity, and healthy weight during pregnancy and the first eighteen months postpartum. The views of both women and PHCPs are reported along with implications for content and format of the subsequent clinically endorsed* Ngala Healthy You, Healthy Baby* (HYHB) website and smartphone app. The paper also reports implementation of the resource and mothers' usage in the first year since its inauguration.

## 2. Method

**Figure 1:**
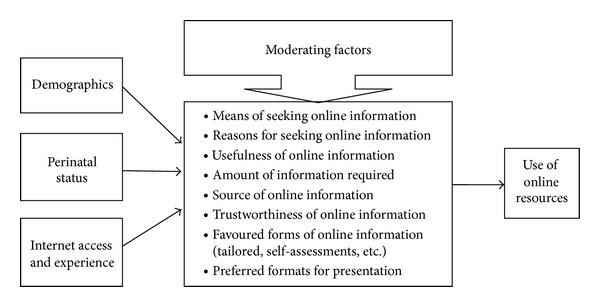
Theoretical framework to review the use of online healthy lifestyle information.

A logic model for this study was developed following an extensive literature review of models and instruments used to explore online health information retrieval and use among parents and health providers [15–18, 37–41] (see Figure 1). The logic model was used to guide development of research instruments and interpretation of results.

Data collection to address the aims of the study included the following: intercept interviews with 53 pregnant women attending hospital antenatal clinics in Western Australia (WA); 12 focus groups with a total of 67 postnatal mothers attending WA urban and rural playgroups; and intercept interviews with 76 PHCPs to determine what information they felt mothers should receive. Reflecting the logic model, the interviews/focus groups sought information on maternal use of online devices and resources for lifestyle information during pregnancy and early postnatal months, identification of main online resources used to seek information, usefulness of online information, amount/format of information required, source of online information sought, trustworthiness of online information, gaps in useful forms of online information (e.g., tailored, self-assessments), and preferred formats for presentation. The interviews and focus groups were audio-taped, subsequently transcribed verbatim, and then analysed using logico-inductive analysis.

Based on the parents' preferences for the content and format of online resources that emerged from the interviews and focus groups, a “*Gap Analysis Scale*” was developed. This was used assess the strengths and weaknesses of existing online resources used by the antenatal and postnatal mothers. Content criteria specified inclusion of online information related to healthy maternal lifestyle (nutrition, activity, and weight), healthy parental lifestyle and modelling to children, emotional wellbeing, and parenting advice, as well as quantity and quality of information. Format criteria included potential to impact on family behaviour to prevent obesity, user-engagement, personalised and tailored information, and local connectedness. Australian websites were located using general search terms (e.g., overweight, obesity, pregnant women, newborn, parents, online programs, healthy lifestyles, nutrition, physical activity, and weight). To apply the gap analysis scale two researchers independently assessed and scored government, nongovernment, and private enterprise websites aimed at pregnant women and new mothers.

The findings of the interviews/focus groups with parents and PHCPs, together with the results of the gap analysis, were presented to key WA government and nongovernment stakeholders and health practitioners to determine how best to overcome existing gaps to reach parents through online resources. Their decision was guided by findings of the interviews/focus groups and gap analysis, together with their need to address staff shortages and capitalise on high online information seeking behaviour of expectant and new parents [16, 17, 27]. The outcome was development of a clinically endorsed* Ngala Healthy You, Healthy Baby *(HYHB) website and app (http://www.ngala.com.au/hyhb). The resources were launched with media coverage and mail-out of information, clinic posters, and patient leaflets to antenatal and child health service providers. Support from the WA Department of Health allowed GPs, obstetricians, midwives, and child health nurses to refer mothers to use the website and app. The web address and app were promoted through maternal and child health networks and newsletters.

One year after the launch of the HYHB website and app in June 2012, resource usage data obtained from Google Analytics were analysed and demographic information of users compared with WA birth statistics [42]. Postcode information accessed from the app was used to determine user demographics in terms of geographic distribution across Australian Bureau of Statistics Statistical Divisions and quintiles of social disadvantage expressed as Socioeconomic Index for Areas (SEIFA) [43] compared to annual births in WA [42].

## 3. Results

### 3.1. Mothers and PHCPs Online Needs and Preferences

In line with recent data [25], the majority of perinatal women interviewed considered online resources their primary source of lifestyle information. Women frequently searched online for information about pregnancy and early childhood, especially those mothers with their first child. The key reason for using the internet was to find short, quick factual answers to immediate concerns, both to confirm their current knowledge and/or to provide reassurance that their issues were normal, without having to visit the doctor for nonmedical reasons. In particular, women wanted information on issues like nutrition and diet, exercise, managing weight gain, sleeping problems, emotional fluctuations, allergies, breastfeeding, and gestational diabetes. They emphasised their need for realistic exercise they could do given their new situation and quick, cheap, safe recipes they could easily prepare. They also wanted information for their partners, as well as information on events and resources in their local area. Mothers highlighted that, contrary to much of current information provided, they wanted hands-on “parent-focused” rather than “child-focused” interactive materials including personalised learning activities that would assist them to become role-models for healthier family lifestyles.

Women wanted credible, evidence-based information, yet few of the women interviewed were satisfied with online information currently available, with many being extremely dissatisfied. While online resources saved time, few users found them trustworthy; many found information contradictory, and Google search terms repeatedly directed them to international websites with no local information. While they trusted government or university websites highly and questioned the trustworthiness of commercial websites, they admitted commercial sites and forums were more accessible and user-friendly with attractive promotions and prizes. Women were not particularly loyal to any healthy lifestyle online resource but instead checked and compared information from numerous sites. When messages conflicted, women usually sought clarification from experienced friends or family members rather than seeking advice from health professionals. Cost and inconvenience were barriers to women visiting a doctor or health professional about daily concerns for which they just wanted quick easy answers, but nearly all those interviewed said they would greatly appreciate their PHCP recommending an online resource with all information in one place and which they could trust.

In terms of the format of online data, perinatal mothers indicated they wanted information relevant to their individual issues, through user self-assessment tools, ongoing tracking of their progress, smartphone apps they could use anywhere any time, instructional video clips, monthly updates on local events and activities in their area, e-newsletters, and information tailored to the developmental stage of their child.

In addition to perinatal mothers' views, PHCPs emphasised the need for positive messages to women about adopting healthy lifestyle changes at this crucial time in life to reduce potential complications and risks for themselves and their child. PHCPs also focused on the need to recommend simple, inexpensive foods and activities both mothers and their children could have at different stages across the perinatal and postnatal periods (Table 1).

### 3.2. Gaps in Online Resources

Gap analysis of websites most frequently used by parents showed government websites scored highly for quality and quantity of information, yet scored low on parent focused, user-friendly advice; recommendations on how to achieve healthy gestational weight gain; and links to services available in their local communities (Table 2). In contrast, private enterprise websites had a lower level of trustworthy/quality information but high levels of user engagement and detailed information on perinatal health needs of mothers. Results for nongovernment organisation websites varied but generally provided medium to relatively high quantity and quality of relevant online information and higher levels of user engagement than government websites but not as high as those of private enterprises. Some of the not-for-profit organisation websites provided information on local activities and ways parents can connect, although with limited reach across regions in WA. Whilst some websites reviewed provided information relevant to stage of pregnancy, none met criteria for personalised, tailored information to address individual needs. Moreover, with the exception of one private website that was not clinically endorsed, none provided interactive information that enabled self-assessment, personalised advice, and tracking of progress, nor did any websites have links to apps which mothers indicated they found convenient and liked using on their mobile phones. The gap analysis also found variable content topics, with limited or no information on paternal lifestyle behaviour, recommended weight gain/loss, or physical activity type and intensity at different stages of pregnancy and postpartum.

### 3.3. Development of a Tailored, Personalised Online Resource

Based on recommendations of key stakeholders, the* Healthy You, Healthy Baby* (HYHB) web information and app were integrated into the website of a leading WA early childhood parent support organisation Ngala (http://www.ngala.com.au). This website was well used and already confidently advocated to parents by GPs, obstetricians, midwives, child health nurses, and child care providers. The content is clinically endorsed by an advisory committee of PHCPs. The HYHB web information was designed to address current gaps and provide perinatal women with convenient access to brief factual information on nutrition, physical activity, weight, emotions, social life, and sleeping patterns, all identified by mothers as key areas of interest. The accompanying app was developed with a self-assessment tool to track maternal lifestyle behaviours and weight during pregnancy and the first 18 months of motherhood. This self-assessment tool was designed to generate supportive tailored feedback and tips on how to make improvements. To sign in, users entered their postcode, thus providing information on their geographic location. Information on their height, initial weight, and stage of pregnancy or postpartum was also collected to allow appropriate feedback and tailored information to be provided.

### 3.4. App and Website Overall Usage

Resource usage data obtained from Google Analytics (http://www.google.com.au/, verified 16 July 2013) showed 14,023 views of HYHB antenatal website pages and 7,596 of postnatal pages over the first year. A total of 2,378 users signed up to the HYHB app over the same time period. With an estimated 33,000 WA births in 2012-13 and 40% of these being first time mothers [42], this extrapolates to 7% of pregnant WA women and 18% of first time mothers using the app. Website views and app new user sign up rates increased from the first six-month period to the second six-month period, with website views increasing to 72% and 14% in the antenatal and postnatal periods, respectively, and app user growth of 47% (62 new users per week increasing to 91 new users per week).

**Figure 2:**
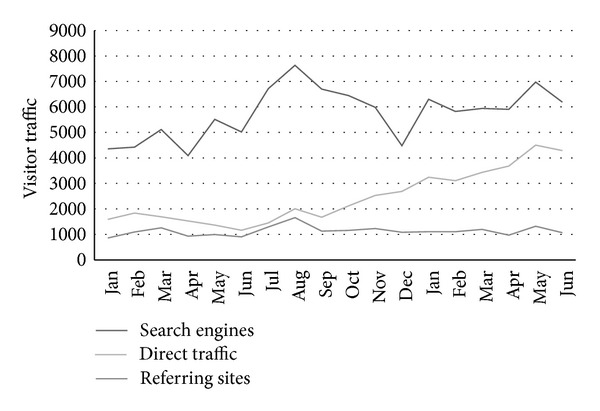
Visitor traffic to www.ngala.com.au from search engines, direct address, and referring sites (January 2012–June 2013).

Traffic to the Ngala website increased significantly after the launch of the HYHB web pages and app (Figure 2). Peaks in website traffic occurred directly after the launch and newspaper media coverage in July 2012, while a large increase in new app users was seen after newspaper publicity in May 2013. Direct traffic has increased steadily since release of the HYHB resource. One quarter of website traffic was directly referred from the app.

### 3.5. App Self-Assessment and Website Content Usage

**Figure 3:**
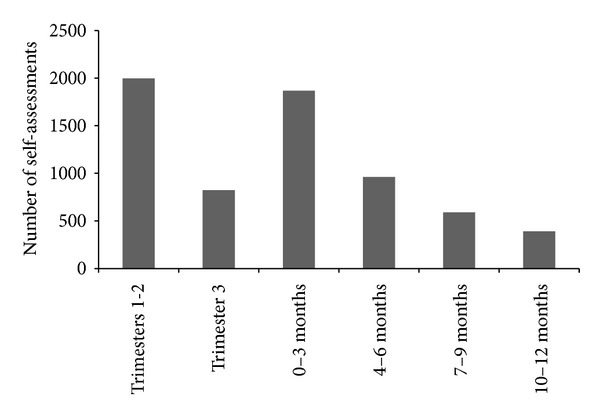
*Ngala Healthy You, Healthy Baby* app self-assessments during the first year of release according to perinatal stage.

HYHB app self-assessments were completed at a rate of 167 per week, with the average person completing 3.6 questionnaires. The highest HYHB app self-assessment usage was in the first two trimesters of pregnancy and in the first 3 months after birth (Figure 3).

**Figure 4:**
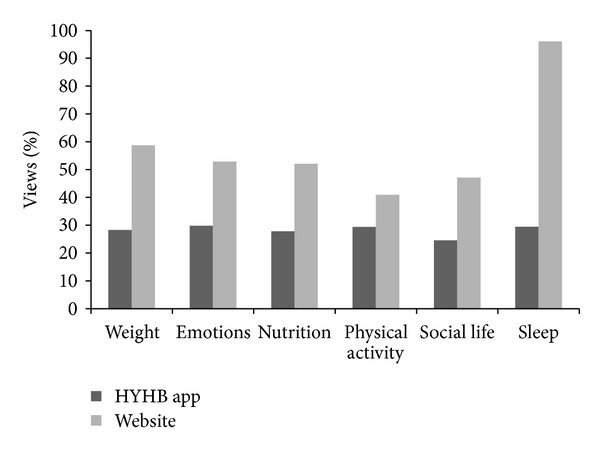
Topics of* Ngala Healthy You, Healthy Baby* app self-assessments and antenatal and postnatal website views.

The most popular HYHB app self-assessments completed over the year were weight (25%) and sleep (18%), followed by nutrition (15%), physical activity (15%), emotions (13%), and social life (13%) (Figure 4). Aside from weight assessment, assessment topics were in similar proportions across the pregnancy but with increasing emphasis on sleep until 9 months postpartum. The highest website page views related to the antenatal period featured nutrition content (40% of views) followed by weight (33%), physical activity (14%), sleep (6%), emotions (5%), and social life (2%) (Figure 4). For page views related to the postnatal period and compared to antenatal, nutrition was again the highest focus (49% of views), with less views for weight (9%) and similar views for physical activity (16%), sleep (11%), emotions (11%), and social life (5%) (Figure 4).

### 3.6. Regional and SEIFA Demographics of Users

When compared to the geographic distribution of mothers' residence for annual births [42], WA regional use of the HYHB app was about 10% lower than in Perth but otherwise showed a similar distribution across regions (Table 3). App users were resident in areas across all quintiles of social disadvantage but were over represented in areas of lower disadvantage compared to distribution of WA births (Table 4).

## 4. Discussion

Mothers are identified as crucial agents of change for prevention of obesity in their offspring [3]. Pregnancy is a critical metabolic period and a time when mothers are most likely to be receptive to changing their lifestyles for the sake of their child [44]. Young women are primary users of internet technology and this research confirmed pregnant women and new mothers are seeking lifestyle advice online during their childbearing years. Women in our research wanted short, quick answers to their pregnancy and child rearing concerns, self-assessment tools, and practical suggestions to achieve change at the family level. They also expressed preference for information readily accessed on mobile devices. Use of online resources and particularly mobile devices shows significant promise for supporting health behaviour change [29, 32, 33, 45]. Not only do online resources provide users with access to health information, but they also enable interactive use, with ongoing collection of personalised data and cueing of the user's behavioural information according to their goals and stages of behaviour change. In this way, individual messages can be tailored to the user's needs while directing them to suitable support [31, 34]. Research shows that such personalised online information with tailored messages is more effective in bringing about behaviour change than static information [32–34]. Likewise, evidence suggests longer-lasting behaviour change since interactive components enhance the user's experience and support their achievement of behavioural goals [29].

The HYHB resources were designed to address the findings of our research that highlighted both young women's needs and the existing gaps in available online resources. Specifically it sought to provide perinatal mothers with a personalised, interactive tailored resource with parent focused, brief advice relevant to their stage of pregnancy and lifestyle assessment, goal setting, and monitoring. The content was clinically endorsed and available via a mobile phone app with similar as well as more detailed factual, practical, and localised information on the website. These combined features desired by women were not currently available on any existing websites.

Monitoring HYHB resource usage in the first year of implementation showed online resource use varied with content, format, and delivery mode. Whilst website hits were higher than new app signups, hits represent both new and repeated visits to the website. However a significant proportion (25%) of website traffic came directly from the app and the rate was increasing, indicating mobile devices are an important tool to engage women. Also, the content accessed varied between app and website information. Weight assessment was most common on the app (25% of assessments) with fairly stable assessment of other lifestyle categories (13–18%). Weight was also an important focus of antenatal website views (34%) but nutrition was the primary topic of webpage views related both to antenatal (40%) and postnatal periods (49%). Views for other topics were significantly lower and variable (5–17%). These results suggest that the simple format of the app leads many users to complete all self-assessments at the same time, but with more frequent focus or return to the weight assessment. In contrast website users may be accessing the website to gain particular information (e.g., nutrition) and may not be exposed to the other categories. Overall the results highlight the value of providing both a website and an app to engage women and to provide supportive information.

Given the magnitude of information in the online environment, promotion of the HYHB resources is a critical aspect of their successful adoption by women. Women in our research and other studies [16, 17] expressed a clear preference for quality assured resources recommended by a trusted health professional. Policy makers and PHCPs guiding development of the online resources also emphasised the need for “clinically endorsed” information that GPs, obstetricians, midwives, child health nurses, and child care providers could confidently refer to women [45]. The credibility of the HYHB resources, web address, and app was promoted through maternal and child health networks and newsletters. Usage data showing steady increase in direct entry to the website URL indicated promotion of this address via health professionals enhanced mothers' use of it. Whilst significant peaks in direct entry to the website and use of the app also coincided with modest short term newspaper promotion, this form of promotion was not sustainable and provided little advantage over promotion through PHCPs. These results highlight the value of engaging health professionals in targeted promotion of healthy lifestyle resources to pregnant women.

For health care services, provision of online health information is identified as a cost-effective approach to reaching broad audiences in many locations [29, 31]. Yet demographic barriers for new parents have been identified [12, 20]. Whilst demographic data for HYHB webpage views is not available, registrations for the app showed slightly higher use in urban than rural areas. This is not unexpected since some WA rural areas have poor internet access or limited PHCPs to refer the app. Of greater significance is lower sign up to the app in areas of higher social disadvantage (Table 4). This may be due to a range of factors including lower access to internet and smartphones, cultural and language barriers to app referral and content, and lack of motivation to achieve healthier lifestyles [22, 24]. Whilst these factors need to be explored, the finding should not distract from significant and increasing uptake of the resource within the first year and the low cost of ongoing implementation through referral by PHCPs.

Whilst many pregnancy-focused websites and apps are available, the strengths of the HYHB resource include attention to women's expressed needs as well as effective behaviour change strategies including self-assessment, goal setting, tailored advice, and feedback. Provision of clinically endorsed information also provides reassurance to PHCPs engaged in implementation. In addition, our formative research identified gaps in useful online lifestyle information including appropriate weight gain during pregnancy and the types and amounts of physical activity considered safe during pregnancy. When addressed by the HYHB resources this information about weight particularly was amongst the highest accessed.

## 5. Conclusions

Online communication is an effective approach to reach pregnant women and new mothers seeking advice about healthy lifestyle and weight management. Mobile phone apps can help to engage women, whereas websites are an accepted source of detailed information. Together they are complementary and if recommended by trusted PHCPs can enhance information retrieval intended to promote maternal healthy lifestyle behaviour change in the perinatal period. Referral of evidence-based online resources by PHCPs provides a low-cost intervention across most geographic and socioeconomic strata without additional demands on health service staff.

## Figures and Tables

**Table 1:** Perinatal women and PHCP online needs and preferences for health lifestyle information.

Perinatal mother's online needs	Primary health care providers' (PHCPs) online needs
Content (i) Factual practical information on one central website (ii) Q&As to immediate concerns without having to wait/pay for a doctor's appointment (iii) Information on parent rather than child focused issues (iv) Reassurance about normality of concerns (v) Information on healthy diets, safe foods, and quick low-cost recipes (vi) Tips to control their weight (vii) Tips on safe and realistic exercise (viii) Information for their partners (ix) Details of support and social activities available in their local area	Content (i) Positive information for parents on what they can do not what they should do (ii) Importance of adopting lifestyle changes, listing benefits to parents and child, and potential complications/risks if not adopted (iii) Information about healthy eating options, for example, intake of sugary drinks (iv) Outline of diets that are easy to achieve and not too expensive (v) Role of depression and obesity and how to recognise and manage these (vi) What foods should be given to children and in what amounts (vii) Ways to incorporate recommendations into their busy and demanding lifestyles (viii) Local information on what support is available and where to go for this (ix) Links to support (x) Monthly newsletters with links to further reading
Format (i) Friendly but professional writing style (ii) Easy to read and navigate (iii) Basic information with links to further reading (iv) Self-assessment quizzes with relevant advice and ideas to support change (v) Information presented according to stages of pregnancy or child development (vi) Noninvasive followup via email, app alerts, or SMS (vii) Forums and blogs but limited by reliability of peer information and time commitment	Format (i) Easy and fun to read (ii) Information that is quick to upload (iii) Use language, icons, animations, photos, and graphics that would attract mothers (iv) Interactive website information

**Table 2:** Outcomes of gap analysis study showing levels of information provided by types of websites most frequently used by parents.

Criteria for assessment	Website type		
	Government *n* = 2	Private *n* = 6	Not-for-profit *n* = 5
Parent focused information	Low	Low/Medium	Low
User friendly format	Low	High	Low
Quality of information	High	Medium	Medium
Use of personalised and tailored information	None	Low	None

**Table 3:** Number and percentage of app users and percentage of Western Australian births per year by mother's region of residence.

	Number of app users in past year	Percentage of all WA users	Regional residence as % of all WA births in 2011 (*n* = 32.259)
Perth	1965	82.9	73.2
Wheatbelt	24	1.0	3.1
Midwest	44	1.9	2.1
South West	146	6.2	9.9
Pilbara	56	2.4	3.1
Kimberley	29	1.2	2.3
Goldfields	43	1.8	3.0
Great Southern	64	2.7	3.1

Total WA users	2371	100.0	100

**Table 4:** Number and percentage of app users and percentage of Western Australian births by SEIFA.

SEIFA level*	Number of app users in past year (*n* = 2.337)	Percentage of all WA users	SEIFA as % of all WA births in 2010 (*n* = 30.568)
I	968	41.4	28.8
II	610	26.1	22.2
III	402	17.2	24.8
IV	237	10.1	15.1
V	120	5.1	9.0

*“I” indicates the 20% of women who are least disadvantaged, whereas “V” represents the highest disadvantage score.
